# Comparison of Heart Team vs Interventional Cardiologist Recommendations for the Treatment of Patients With Multivessel Coronary Artery Disease

**DOI:** 10.1001/jamanetworkopen.2020.12749

**Published:** 2020-08-10

**Authors:** Michael B. Tsang, J. D. Schwalm, Sumeet Gandhi, Matthew G. Sibbald, Amiram Gafni, Mathew Mercuri, Omid Salehian, Andre Lamy, Dan Pericak, Sanjit Jolly, Tej Sheth, Craig Ainsworth, James Velianou, Nicholas Valettas, Shamir Mehta, Natalia Pinilla, Bobby Yanagawa, Li Zhang, Victor Chu, Dominic Parry, Richard Whitlock, Adel Dyub, Irene Cybulsky, Lloyd Semelhago, Kostas Ioannou, Adnan Hameed, Douglas Wright, Amin Mulji, Saeed Darvish-Kazem, Nandini Gupta, Ahmed Alshatti, Madhu K. Natarajan

**Affiliations:** 1 Division of Cardiology, Department of Medicine, McMaster University, Hamilton, Ontario, Canada; 2 Division of Cardiology, Department of Medicine, University of Toronto, Toronto, Ontario, Canada; 3 Trillium Health Partners, Mississauga, Ontario, Canada; 4 Center for Health Economics and Policy Analysis, Department of Health Research Methods, Evidence and Impact, McMaster University, Hamilton, Ontario, Canada; 5 Division of Cardiovascular Surgery, Department of Surgery, McMaster University, Hamilton, Ontario, Canada; 6 Division of Cardiovascular Surgery, St Michael’s Hospital, Toronto, Ontario, Canada; 7 Division of Cardiac Surgery, Department of Surgery, University of Toronto, Toronto, Ontario, Canada; 8 Dunedin Hospital, Otago, New Zealand; 9 Oakville Trafalgar Memorial Hospital, Oakville, Ontario, Canada; 10 Brampton Civic Hospital, William Osler Health System, Brampton, Ontario, Canada

## Abstract

**Importance:**

Although the heart team approach is recommended in revascularization guidelines, the frequency with which heart team decisions differ from those of the original treating interventional cardiologist is unknown.

**Objective:**

To examine the difference in decisions between the heart team and the original treating interventional cardiologist for the treatment of patients with multivessel coronary artery disease.

**Design, Setting, and Participants:**

In this cross-sectional study, 245 consecutive patients with multivessel coronary artery disease were recruited from 1 high-volume tertiary care referral center (185 patients were enrolled through a screening process, and 60 patients were retrospectively enrolled from the center’s database). A total of 237 patients were included in the final virtual heart team analysis. Treatment decisions (which comprised coronary artery bypass grafting, percutaneous coronary intervention, and medication therapy) were made by the original treating interventional cardiologists between March 15, 2012, and October 20, 2014. These decisions were then compared with pooled-majority treatment decisions made by 8 blinded heart teams using structured online case presentations between October 1, 2017, and October 15, 2018. The randomized members of the heart teams comprised experts from 3 domains, with each team containing 1 noninvasive cardiologist, 1 interventional cardiologist, and 1 cardiovascular surgeon. Cases in which all 3 of the heart team members disagreed and cases in which procedural discordance occurred (eg, 2 members chose coronary artery bypass grafting and 1 member chose percutaneous coronary intervention) were discussed in a face-to-face heart team review in October 2018 to obtain pooled-majority decisions. Data were analyzed from May 6, 2019, to April 22, 2020.

**Main Outcomes and Measures:**

The Cohen κ coefficient between the treatment recommendation from the heart team and the treatment recommendation from the original treating interventional cardiologist.

**Results:**

Among 234 of 237 patients (98.7%) in the analysis for whom complete data were available, the mean (SD) age was 67.8 (10.9) years; 176 patients (75.2%) were male, and 191 patients (81.4%) had stenosis in 3 epicardial coronary vessels. A total of 71 differences (30.3%; 95% CI, 24.5%-36.7%) in treatment decisions between the heart team and the original treating interventional cardiologist occurred, with a Cohen κ of 0.478 (95% CI, 0.336-0.540; *P* = .006). The heart team decision was more frequently unanimous when it was concordant with the decision of the original treating interventional cardiologist (109 of 163 cases [66.9%]) compared with when it was discordant (28 of 71 cases [39.4%]; *P* < .001). When the heart team agreed with the original treatment decision, there was more agreement between the heart team interventional cardiologist and the original treating interventional cardiologist (138 of 163 cases [84.7%]) compared with when the heart team disagreed with the original treatment decision (14 of 71 cases [19.7%]); *P* < .001). Those with an original treatment of coronary artery bypass grafting, percutaneous coronary intervention, and medication therapy, 32 of 148 patients [22.3%], 32 of 71 patients [45.1%], and 6 of 15 patients [40.0%], respectively, received a different treatment recommendation from the heart team than the original treating interventional cardiologist; the difference across the 3 groups was statistically significant (*P* = .002).

**Conclusions and Relevance:**

The heart team’s recommended treatment for patients with multivessel coronary artery disease differed from that of the original treating interventional cardiologist in up to 30% of cases. This subset of cases was associated with a lower frequency of unanimous decisions within the heart team and less concordance between the interventional cardiologists; discordance was more frequent when percutaneous coronary intervention or medication therapy were considered. Further research is needed to evaluate whether heart team decisions are associated with improvements in outcomes and, if so, how to identify patients for whom the heart team approach would be beneficial.

## Key Points

**Question:**

Do treatments recommended by a heart team differ from those recommended by an original treating interventional cardiologist for patients with multivessel coronary artery disease?

**Findings:**

In this cross-sectional study of 245 patients with multivessel coronary artery disease, heart team treatment recommendations indicated moderate agreement (with discordance in 30% of cases) with the original treating interventional cardiologist. Unanimous decisions within the heart team and agreement between the heart team interventional cardiologist and the original treating interventional cardiologist were less frequent in this subset of cases, while interventional cardiologist recommendations for angioplasty and medication therapy were more frequent.

**Meaning:**

The study’s findings indicated that heart team recommendations differed from those of the original treating interventional cardiologist in approximately one-third of cases; this subset of cases was associated with a greater number of divergent opinions between interventional cardiologists and within the heart team.

## Teaser

This cross-sectional study examines the differences in treatment decisions between a heart team and an original treating interventional cardiologist for patients with multivessel coronary artery disease.

## Introduction

In guidelines for revascularization, the heart team model has been given the highest level of recommendation (class 1 in the American College of Cardiology/American Heart Association guidelines) for treatment decision-making in patients with complex multivessel coronary artery disease (CAD); however, this recommendation was primarily based on consensus opinion (considered level C evidence in the American College of Cardiology/American Heart Association guidelines).^1,2,3^ The goal of a heart team is to use multidisciplinary expertise in decision-making for the treatment of patients with complex conditions. Although the membership of a heart team can vary, it generally includes an interventional cardiologist, a cardiovascular surgeon, and a noninvasive cardiologist. Although, to our knowledge, no randomized clinical trials have been conducted to evaluate the benefits of the heart team approach with regard to decision-making or outcomes, observational data suggest that heart team–derived management decisions are safe, and the implementation of heart team decision-making is associated with improvements in patient outcomes.^4,5,6^ Furthermore, the use of group decision-making, commonly referred to as collective intelligence, has been associated with improvements in decision-making in multiple settings.^7,8,9,10,11^

Heart team decisions for patients with multivessel CAD also have face validity. Decisions by heart teams have been reported to be feasible, reproducible, and reasonably concordant with the Appropriate Use Criteria for Coronary Revascularization.^4,12,13,14^ Multivessel CAD is a complex condition with multiple layers of interacting variables, including objective anatomical data from angiography, functional data, clinical data (symptoms and comorbidities), sociodemographic variables, and patient values and goals. Often, variables within the same patient may indicate competing treatment strategies. The expertise of individual physicians or surgeons is specific to their professional training and experience.^15^ Hence, the use of multiple perspectives may balance competing variables and reduce potential specialty-associated biases.^16^

However, implementation of the heart team approach is resource intensive.^14^ It requires coordination of multiple schedules, administrative infrastructure to collate and organize data, determination of standardized risk scores (SYNTAX score [developed in the Synergy Between PCI (percutaneous coronary intervention) With Taxus and Cardiac Surgery, or SYNTAX, clinical trial], European System for Cardiac Operative Risk Evaluation [EuroSCORE] score, and Society of Thoracic Surgeons [STS] score), coordination of case information by central triage, and communication of consensus decisions to referring physicians.^17^ As such, it can be challenging to integrate a heart team into the workflow of high-volume medical centers.^17^ Furthermore, time-sensitive decisions for acute presentations may be challenging to coordinate. Hence, it is important to assess whether heart team decision-making would be any different than existing decision-making structures. The extent of difference between the decision-making of the individual physician and the heart team is currently unknown.

Previous studies have indicated that, in patients with multivessel CAD, the treatment decision recommended by the original treating interventional cardiologist is the best indicator of the final treatment received.^18^ We sought to examine the agreement between the original treating interventional cardiologist and the heart team regarding treatment decisions for patients with multivessel CAD.

## Methods

This study was approved by the Hamilton Integrated Research Ethics Board with a waiver of informed consent for patients because the study presented a low risk to them. All physicians and surgeons involved in the study provided written consent. The Strengthening the Reporting of Observational Studies in Epidemiology (STROBE) reporting guideline was used for this study.

### Patient Population and Recruitment

Multivessel CAD was defined as (1) stenosis of 70% or more in 3 epicardial coronary vessels or stenosis of at least 1.5 mm in their branches or (2) stenosis in 2 epicardial coronary vessels with involvement of the proximal left anterior descending artery.^19,20^ Patients with stable CAD and patients with presentations of unstable angina or non–ST-segment elevation myocardial infarction were included. Patients who had acute ST-segment elevation myocardial infarction, who were hemodynamically unstable, or who had a clear independent indication for cardiac surgery (eg, severe aortic stenosis or left main stenosis of ≥50%) were excluded.

**Figure 1.**
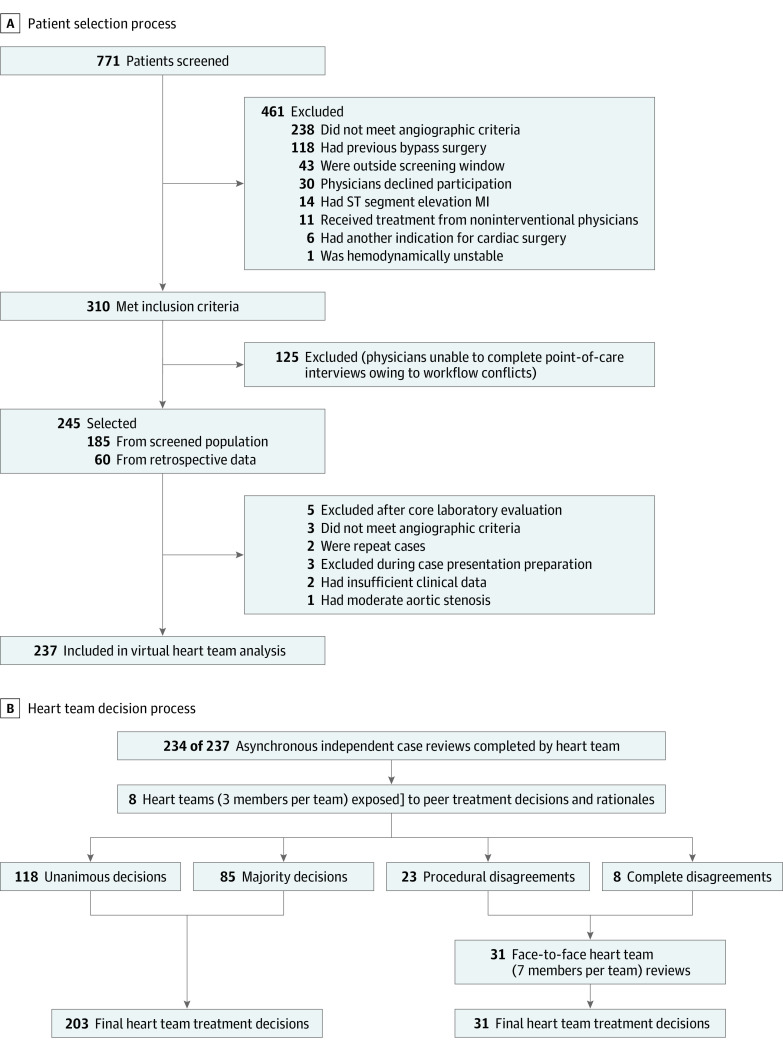
Patient Selection and Heart Team Decision Flowcharts MI indicates myocardial infarction. A, Patient selection process. B, Heart team decision process. Unanimous decisions were those in which all 3 of the team members arrived at the same decision. Majority decisions were those in which 2 of 3 team members made the same decision. Procedural disagreements were those in which 2 team members chose a procedural treatment and 1 member disagreed with that treatment. Complete disagreements were those in which all 3 of the team members arrived at different treatment decisions. Face-to-face heart team reviews were held only when procedural disagreements or complete disagreements with the heart team occurred.

A total of 771 patients who had multivessel CAD between July 17, 2012, and October 20, 2014 were screened at 1 high-volume tertiary care referral center (Figure 1A). Of those, 310 patients were eligible for participation; 125 of those patients were excluded because the original treating interventional cardiologist was unable to complete the interview owing to time constraints. For the remaining 185 patients, the actual treatments received and the most important factors underlying the original treatment decisions were documented at the time of the angiogram through a questionnaire administered by our research assistants. An additional 60 patients with approximately the same distribution of original treatment recommendations (CABG [coronary artery bypass grafting], PCI, or medication therapy) from March 15 to August 3, 2012, were consecutively retrieved from our center’s database (using *multivessel coronary artery disease* as the search term) to reach our final sample of 245 patients. Because there was no formal heart team at our center at the time of this study, all treatment decisions (even those for cases that were retrospectively recruited) were those of the individual interventional cardiologist at the time of the angiogram. Five additional patients were excluded after the core laboratory review, and 3 patients were excluded based on clinical criteria during preparation for the final case presentation via the virtual heart team interface, resulting in 237 patients included in the virtual heart team analysis. The flowchart for case inclusion in the final heart team analysis is detailed in Figure 1B.

### Interventional Cardiologists and Heart Team

Almost all of the original treating interventional cardiologists had more than 10 years of experience, with 1 cardiologist having between 5 and 10 years of experience. Each interventional cardiologist performed 750 diagnostic angiograms, 250 to 300 diagnostic PCIs, and 90 to 100 primary PCIs annually.

Clinical summaries, diagnostic angiogram (dynamic cine) images, SYNTAX scores (which calculate CAD complexity, with higher scores indicating greater complexity),^21^ STS scores (which estimate the risk of morbidity and mortality after surgery, with higher scores indicating higher risk),^22,23,24^ and EuroSCOREs (which calculate the risk of mortality after cardiac surgery, with higher scores indicating higher risk)^22,23,25^ were collated by 2 senior cardiology trainees (S.G. and N.G.) and presented via a virtual heart team interface. Clinical summaries included all medical histories, medications, physical examination results (including body mass index [calculated as weight in kilograms divided by height in meters squared]), and social histories (including occupation, living situation, social support, level of independence with activities of daily living, and mobility), laboratory findings, 12-lead electrocardiogram results, noninvasive stress testing results, and echocardiogram results. These summaries were obtained through a medical record review by the cardiology trainees and presented using a structured template (eMethods in the Supplement). All SYNTAX scores were calculated by 2 interventional cardiologists (M.B.T. and M.G.S.) using core laboratory software (QAngio XA; Medis); the 2 cardiologists were blinded to the original treatment assignments.

Eight 3-member heart teams with randomized membership (each containing 1 interventional cardiologist, 1 cardiovascular surgeon, and 1 noninvasive cardiologist) independently evaluated 237 cases through the virtual heart team interface using structured online case presentations and cine angiogram images of patients with multivessel CAD. Cases were randomized into 6 sets of 5 cases each (approximately 30 cases per heart team) using a stratified randomization procedure to ensure relatively equal heart team exposure to case complexity and a similar ratio of original treatment strategies (CABG, PCI, and medication therapy).

Each heart team member’s decision was initially made asynchronously and independently, with the member blinded to other team members, the decisions of other team members, and the decision of the original treating interventional cardiologist. The decisions of individual heart team members and the 3 primary reasons for each of their decisions were acquired between October 1, 2017, and October 30, 2018. The heart team members had access to the responses of the other heart team members only after all members had submitted their independent decisions; a change in decision was then allowed.

### Comparison and Main Outcomes

Interrater reliability between the heart team and the original treating interventional cardiologist was measured by the Cohen κ coefficient and the frequency of agreement and disagreement. In all cases, the pooled-majority decision from the heart team was compared with the treatment decision of the original treating interventional cardiologist.

For the initial 3-member heart team online review, either the unanimous decision (all 3 of the heart team members chose the same treatment) or the majority decision (2 of the 3 heart team members chose the same treatment) from the virtual heart team interface was considered the final heart team management decision (Figure 1B). Cases in which all 3 of the heart team members disagreed or in which procedural discordance occurred (eg, 2 members chose CABG, but the surgeon chose medication therapy) were reconciled on a face-to-face basis by a 7-member heart team (3 interventional cardiologists, 2 general cardiologists, and 2 cardiovascular surgeons), which comprised a subset of the entire heart team cohort who volunteered to participate in the process (Figure 1B). After face-to-face discussions, heart team members submitted their decisions independently, using an electronic interface to arrive at a majority decision. The heart team evaluation process for included cases is detailed in Figure 1B.

For the post hoc subgroup analyses, we first stratified the agreement and disagreement by (1) unanimous vs majority decision within the heart team, (2) original treatment recommendation received by the patient, and (3) agreement or disagreement between the heart team interventional cardiologist vs the original treating interventional cardiologist. Second, we assessed the frequency of treatment strategies between the heart team vs the original treating interventional cardiologist and performed the same comparison between the different members of the heart team (noninvasive cardiologist, cardiovascular surgeon, and interventional cardiologist). Third, we performed pairwise comparisons of treatments between experts from different domains (eg, heart team interventional cardiologist vs original treating interventional cardiologist, heart team interventional cardiologist vs heart team cardiovascular surgeon, or heart team interventional cardiologist vs heart team noninvasive cardiologist) to evaluate the extent of agreement. Fourth, we evaluated the number of cases in which patient preference was an important factor in the treatment decision of the original treating interventional cardiologist.

### Sample Size

The goal of the study was to estimate the agreement, as measured by the Cohen κ statistic, between the 2 methods of classifying cases (original treating interventional cardiologist vs heart team). To our knowledge, no estimates of the κ statistic comparing the 2 types of classification have been reported in the literature. Only estimates of agreement between potential heart team members (cardiovascular surgeons, interventional cardiologists, and noninvasive cardiologists) for a series of 6 cases have been reported (κ = 0.44).^16^ Hence, our sample size calculation was based on an acceptable range of precision for the point estimate.

We assumed that for a sample size of 200, allowing for the true value of the interclass κ coefficient to range from 0.286 to 0.792, a 2-sided 95% CI for the interclass κ statistic would extend from the observed value of κ by 0.142 at the lower estimates and by 0.086 at the highest estimate. That assumption was based on an approximate prevalence of CABG ranging from 60% to 70% of the total sample at our center, as derived from our pilot data as well as data from the Variations in Revascularization Practice in Ontario database for a low to medium PCI to CABG ratio, which is the ratio represented by our center.^18^

### Statistical Analysis

The Cohen κ statistic, calculated from the standard 2 by 2 table, was used to examine overall agreement between the heart team and the individual cardiologist regarding the primary and secondary outcomes. The generally accepted levels of agreement based on the κ statistic are as follows: 0.01 to 0.20, indicating slight agreement; 0.21 to 0.40, indicating fair agreement; 0.41 to 0.60, indicating moderate agreement; 0.61 to 0.80, indicating substantial agreement; and 0.81 to 0.99, indicating almost perfect agreement. The strength of agreement, as described by the κ statistic, has been previously defined.^26^ The 95% CI for the κ coefficient was also calculated. Baseline comparisons across the original 3 treatment strategies were statistically compared using an χ^2^ test for categorical variables and an analysis of variance for continuous variables. A 2-tailed *P* < .05 was considered statistically significant. Statistical analyses were performed using SAS software, version 9.2 (SAS Institute Inc). Data were analyzed from May 6, 2019, to April 22, 2020.

## Results

Of the 237 patients included in the heart team analysis, complete data were available for 234 patients (98.7%). Among those, the mean (SD) age was 67.8 (10.9) years; 176 patients (75.2%) were male, and 191 patients (81.4%) had stenosis in 3 epicardial coronary vessels. The baseline patient characteristics are summarized in Table 1. Significant differences were found between patients based on original treatment strategy, including differences in age, diabetes status, cognitive dysfunction, angiogram characteristics, SYNTAX scores, EuroSCOREs, STS scores for mortality, and body mass index (Table 1).

**Table 1.** Baseline Characteristics of Patients Included in Final Analysis Stratified by Original Treatment Recommendation Received

Characteristic	Original treatment, No. (%)				*P* value for 3-way comparison
	Overall (n = 234)	CABG (n = 148)	PCI (n = 71)	Medication therapy (n = 15)	
Age, mean (SD), y	67.8 (10.9)	66.7 (9.7)	68.3 (12.7)	74.9 (10.5)	.02
Male sex	176 (75.2)	116 (78.4)	51 (71.8)	9 (60.0)	.21
Treatment indication					
Stable CAD or angina	97 (41.5)	69 (46.6)	21 (29.6)	7 (46.7)	.18
Unstable angina or non-STEMI	123 (52.6)	69 (46.6)	46 (64.8)	8 (53.3)	
Reperfused STEMI	2 (0.9)	1 (0.7)	1 (1.4)	0	
Ventricular arrhythmia	3 (1.3)	1 (0.7)	2 (2.8)	0	
Cardiomyopathy or CHF	9 (3.8)	8 (5.4)	1 (1.4)	0	
Comorbidities					
Previous MI	38 (16.2)	21 (14.2)	12 (16.9)	5 (33.3)	.16
Diabetes	99 (42.3)	72 (48.6)	21 (29.6)	6 (40)	.03
Renal dysfunction	44 (18.8)	25 (16.9)	16 (22.5)	3 (20.0)	.62
Dialysis	9 (3.8)	8 (5.4)	1 (1.4)	0	.26
COPD	18 (7.7)	9 (6.1)	7 (9.9)	2 (13.3)	.43
Previous stroke	27 (11.5)	16 (10.8)	10 (14.1)	1 (6.7)	.65
Cognitive dysfunction	11 (4.7)	3 (2.0)	7 (9.9)	1 (6.7)	.04
Angiographic characteristics					
3VD	191 (81.6)	133 (89.9)	44 (62.0)	14 (93.3)	<.001
2VD with prox LAD	43 (18.4)	15 (10.1)	27 (38.0)	1 (6.7)	
Test results, mean (SD)					
LV function ejection fraction, %	49.2 (11.2)	48.4 (11.1)	51.3 (11.0)	46.8 (12.4)	.15
BMI	29.6 (6.8)	30.1 (6.0)	29.8 (8.0)	24.1 (5.4)	.005
SYNTAX score	28.6 (10.7)	30.9 (10.4)	23.5 (9.9)	29.2 (9.6)	<.001
EuroSCORE	2.2 (2.4)	1.9 (1.9)	2.4 (2.9)	3.9 (4.0)	.006
STS score					
Mortality	1.6 (1.7)	1.4 (1.5)	1.8 (2.0)	2.8 (1.7)	.005
Morbidity and mortality	12.0 (8.4)	11.8 (7.7)	11.6 (9.5)	17.0 (8.9)	.11

Abbreviations: 2VD with prox LAD, 2 epicardial coronary vessels with involvement of the proximal left anterior descending artery; 3VD, 3 epicardial coronary vessels; BMI, body mass index (calculated as weight in kilograms divided by height in meters squared); CABG, coronary artery bypass grafting; CAD, coronary artery disease; CHF, congestive heart failure; COPD, chronic obstructive pulmonary disease; EuroSCORE, European System for Cardiac Operative Risk Evaluation score; LV, left ventricular; MI, myocardial infarction; PCI, percutaneous coronary intervention; STEMI, ST-segment elevation myocardial infarction; STS, Society of Thoracic Surgeons; SYNTAX, Synergy Between PCI With Taxus and Cardiac Surgery clinical trial.

**Figure 2.**
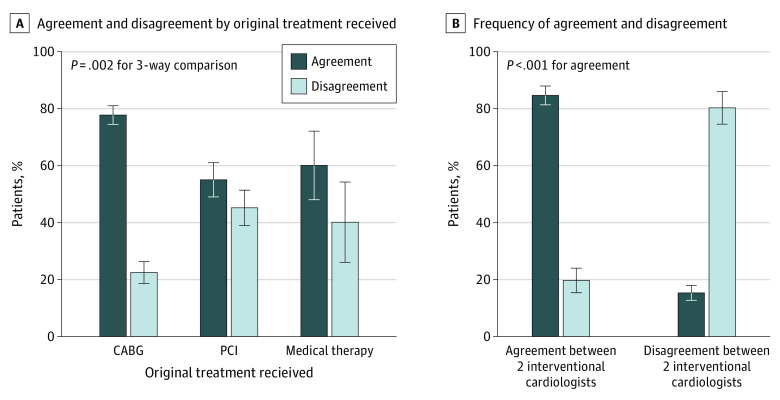
Agreement and Disagreement Between Heart Team and Original Treating Interventional Cardiologist Agreement and disagreement were defined as the concordance or discordance of treatment recommendations between the heart team and the original treating interventional cardiologist. Agreement and disagreement between 2 interventional cardiologists were defined as the concordance or discordance of treatment recommendations between the heart team interventional cardiologist and the original treating interventional cardiologist. Error bars represent SEs. CABG indicates coronary artery bypass grafting and PCI, percutaneous coronary intervention. A, Agreement and disagreement by original treatment received. B, Frequency of agreement and disagreement.

A paired analysis of the original treatment decisions vs the heart team treatment decisions revealed a κ coefficient of 0.478 (95% CI, 0.336-0.540; *P* = .006), which was consistent with moderate agreement (Table 2). This finding was based on 71 differences (30.3%; 95% CI, 24.5%-36.7%) between treatment recommendations made by the heart team and those made by the original treating interventional cardiologist (Table 2). Of those with an original treatment recommendation for CABG, PCI, and medication therapy, 32 of 148 patients (22.3%), 32 of 71 patients (45.1%), and 6 of 15 patients (40.0%), respectively, received a different treatment recommendation from the heart team review than from the original treating interventional cardiologist; this difference across the 3 groups was statistically significant (*P* = .002) (Figure 2A). Of the 234 cases that received a complete Heart Team review, 31 cases (13.2%) required face-to-face reviews owing to complete disagreement between all members (8 of 31 cases) or procedural discordance (23 of 31 cases).

**Table 2.** Paired Analysis Between Heart Team Treatment Decision vs Original Treatment Decision for Individual Cases

Decision	Between heart team and original treating interventional cardiologist				Cohen κ		*P* value
	Agreement	95% CI	Disagreement	95% CI	Value	95% CI	
Heart team treatment vs original treatment, No. (%)	163 (70)	78.2-89.8	71 (30)	11.2-30.7	0.478	0.336-0.540	.006
Cases, No./Total No. (%)							
Unanimous	109/163 (66.9)	59.08-74.04	28/71 (39.4)	28.0-51.8	NA	NA	<.001
Majority	54/163 (33.1)	26.0-40.9	42/71 (60.6)	48.25-71.97	NA	NA	

The heart team decision was more frequently unanimous when it was concordant with the decision of the original treating interventional cardiologist (109 of 163 cases [66.9%]) compared with when it was discordant (28 of 71 cases [39.4%]; *P* < .001) (Table 2). When the heart team agreed with the original treatment decision, the decision of the heart team interventional cardiologist was more frequently in agreement with that of the original treating interventional cardiologist (138 of 163 cases [84.7%]) compared with when the heart team disagreed with the original treatment decision (14 of 71 cases [19.7%]; *P* < .001) (Figure 2B).

**Figure 3.**
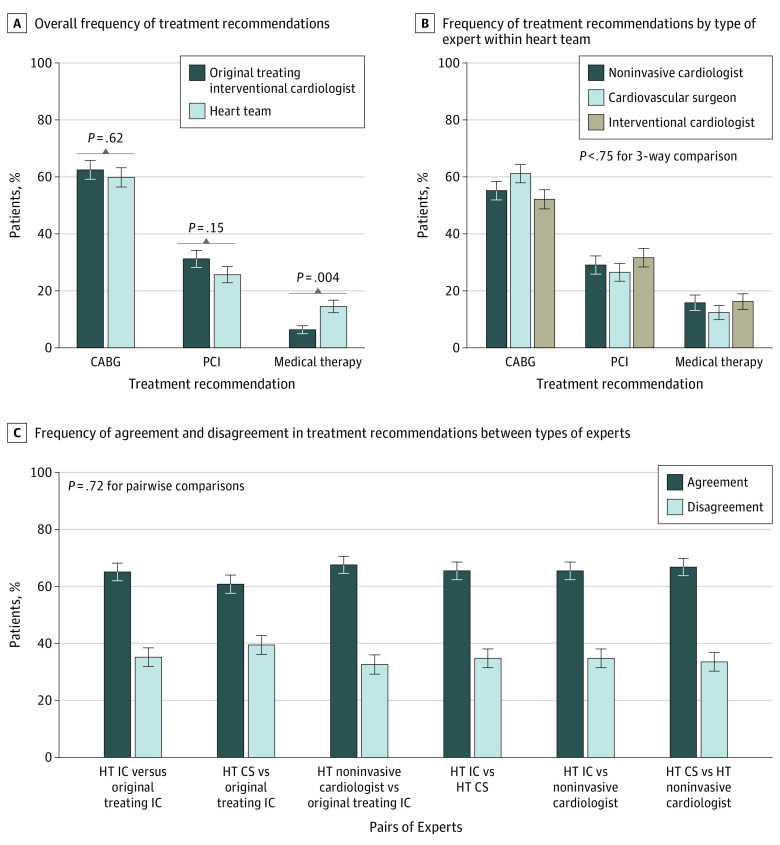
Treatment Recommendations Error bars represent SEs. CABG indicates coronary artery bypass grafting; CS, cardiovascular surgeon; HT, heart team; IC, interventional cardiologist; and PCI, percutaneous coronary intervention. A, Overall frequency of treatment recommendations. B, Frequency of treatment recommendations by type of expert within heart team. C, Frequency of agreement and disagreement in treatment recommendations between types of experts.

The frequency with which the 3 treatment strategies were chosen by the heart team and the original treating interventional cardiologist is represented in Figure 3A. The overall frequency of treatment recommendations was not significantly different between the original treating interventional cardiologist and the heart team for CABG (148 of 237 patients [62.4%] vs 140 of 234 patients [59.8%], respectively; *P* = .62) or PCI (74 of 237 patients [31.2%] vs 60 of 234 patients [25.6%], respectively; *P* = .15) . However, medication therapy was less frequently recommended by the original treating interventional cardiologist than by the heart team (15 of 237 patients [6.3%] vs 34 of 234 patients [14.5%], respectively; *P* = .004).

The frequency with which the different heart team members chose the 3 treatment strategies is summarized in Figure 3B. Individual heart team members did not significantly differ in the frequency with which they selected a particular treatment (eg, CABG was selected for 129 of 234 cases [55.1%] reviewed by noninvasive cardiologists, 143 of 234 cases [61.1%] reviewed by cardiovascular surgeons, and 122 of 234 cases [52.1%] reviewed by interventional cardiologists; *P* = .75). Pairwise comparisons of agreement and disagreement in treatment recommendations between experts from different domains are shown in Figure 3C. The pairwise comparisons were not significantly different (eg, for the pairing of a heart team interventional cardiologist vs an original treating interventional cardiologist, treatment agreement was 152 of 234 cases [65.0%] and treatment disagreement was 82 of 234 cases [35.0%]; for the pairing of a heart team cardiovascular surgeon vs an original treating interventional cardiologist, treatment agreement was 142 of 234 cases [60.7%] and treatment disagreement was 92 of 234 cases [39.3%]; *P* = .72).

Of the 179 screened patients included in the study, patient preference was known in 173 cases (96.6%); in only 16 of 169 cases (8.9%) did the original treating interventional cardiologist indicate that patient preference was an important factor in their treatment decisions. The consideration of patient preference as an important variable in treatment decisions was more frequent in cases in which the heart team agreed with the original treating interventional cardiologist (10 of 179 cases [5.6%]) compared with those in which the heart team disagreed with the original treating interventional cardiologist (6 of 179 cases [3.3%]).

## Discussion

To our knowledge, this cross-sectional study conducted at a high-volume tertiary care center is the first to compare the agreement between treatment decisions made by a heart team with those made by an original treating interventional cardiologist. The heart team model is recommended for the treatment of structural heart interventions and multivessel CAD in cardiology guidelines worldwide.^1,2,3,27,28^ Data regarding the heart team approach, although increasing, are still limited.^5,29^ Furthermore, the extent of difference in the decisions of a heart team compared with those of an individual physician was previously unknown.

At our center, the treatment recommended by the heart team differed from that of the original treating interventional cardiologist in 30.3% of cases. This finding has important practical implications. If heart team recommendations were found to be associated with improvements in outcomes, there may be a subset of patients for whom the heart team approach would be most beneficial. Given the extensive resources required for heart team implementation, selection for this subset of patients may maximize heart team efficiency.

Based on a post hoc analysis, the subset of cases in which the heart team decision differed from the original treatment decision was associated with an increased frequency of discordant treatment decisions at the physician or surgeon level. In cases in which the heart team disagreed with the original treatment decision, unanimous decisions within the heart team were less frequent than in cases in which they agreed with the original decision. This finding suggests that competing viewpoints exist in such cases. Furthermore, disagreement between the heart team and the original treating interventional cardiologist was associated with a more than 4-fold greater disagreement between the heart team interventional cardiologist and the original treating interventional cardiologist. In addition, disagreement between the heart team decision and the original treatment decision was 2-fold greater among patients in whom the original treatment was PCI (45.1%) or medication therapy (40.0%) compared with CABG (22.3%). In patients for whom PCI and medication therapy need to be considered, the balancing of competing risks, benefits, and compromises may be more complex, challenging, or frequent; these dilemmas may also be present but may occur less frequently when CABG is chosen.

Although heart team decisions adhered to the Appropriate Use Criteria for Coronary Revascularization in 99.3% of the cases, 29.2% of patients who received recommendations for PCI had conditions that were categorized as uncertain according to the criteria, and 5.7% of patients had conditions that were categorized as inappropriate.^13^ Because the appropriate use criteria are based on anatomical factors that define prognostic implications, degree of ischemia, presence of symptoms, and baseline medication therapies, they do not capture all variables, such as comorbidity, frailty, life expectancy, surgical risk, patient preference, and social context, which are often important considerations in the final therapeutic decision.^19^

One hypothesized outcome of a heart team review may be that the provision of varying perspectives will help to reconcile context-specific factors during the consideration of multiple treatment options. In this study, the differences between the heart team decisions and the original treatment decisions were not associated with an overall difference in the frequency with which PCI or CABG was recommended. Although the heart team recommended medication therapy with higher frequency than the original treating interventional cardiologist, the numbers were too small to be meaningful. The difference was also not associated with expert domain–specific preferences for particular treatment strategies. Furthermore, paired analyses revealed agreement between different expert domains in approximately two-thirds of cases in all comparisons. This finding suggests that variance in the final heart team decision is equally dependent on all heart team members.

Despite the challenges of addressing uncertain cases, observational studies have indicated that the heart team model for decision-making is feasible and that decisions are implemented in most cases (93%). Heart team decisions are also reproducible 74% to 80% of the time.^4,12,13,14^ Some variability may be justified because definable factors, such as coronary complexity (ie, SYNTAX score), only account for a portion of the clinical decision.^30^

The conflicting treatment decisions observed in uncertain cases of multivessel CAD have been recognized by guidelines, position statements, and clinicians.^1,2,31^ If use of the heart team approach were found to be associated with improvements in outcomes, selection of these cases a priori would likely require a scoring tool that uses common clinical characteristics (eg, age, frailty, cognitive dysfunction, and SYNTAX score) to quantify the therapeutic dilemma. Because multivessel CAD accounts for approximately 25% to 60% of patients with CAD,^18^ such a tool would have wide applicability.

The novel design of the heart team decision-making process underlies the strength of this study. While heart team meetings at most centers are face to face,^14^ the initial online structured case presentation used in this study was essential to answering our study question. First, it resulted in efficient completion (98.7%) of a high volume of cases. The efficiency of this model is also highlighted by the fact that face-to-face meetings to reconcile treatment decisions were necessary in only 13% of the most difficult cases. While the model may still need to evolve, asynchronous aggregate decisions may potentially complement existing heart team operations by facilitating time-sensitive decisions at high-volume centers. Second, the online case presentation served to control for the social factors that can have negative implications for true group decision-making. Social factors can undermine the diversity of input in group decision-making and the benefits of collective intelligence.^32,33^ Furthermore, to answer our study question, the heart team decisions needed to reflect true group decisions rather than the decisions of a few influential individuals.^34,35^ This outcome was accomplished through randomized heart team membership, blinding of heart team members to ensure independent decision-making, and exposure to the input of other heart team members only after independent review.

No data are currently available to inform the structure and function of optimal heart team operations. Hence, our study was guided by the increasing body of empirical data in the cognitive sciences that have guided optimal group decision-making in other settings.^7,8,11,32,36,37,38^ There is a cognitive advantage to maximizing diversity through pooled decision-making.^8,38,39^ The ability for individuals to independently submit their decisions may reduce the momentum bias that the group can have on the individual.^33,40^ The opportunity for heart team members to revise their decisions after independent thought also allows them to consider alternative perspectives. Pooled aggregated decisions that are reached after each team member has had the opportunity to consider alternative viewpoints have been associated with more accurate results compared with pooled independent decisions alone.^36^

### Limitations

This study has several limitations. First, although the study suggests that the heart team approach is associated with positive results, it remains unknown whether this approach is more beneficial than others. Answering this question would require a randomized clinical trial that examined heart team decisions vs individual decisions. Second, heart team decisions were often made independently of patient preferences. When our screened cohort was interviewed, it appeared that patient preference was an important factor in approximately 9% of decisions made by the original treating interventional cardiologist. Third, our study was performed at a single tertiary care referral center and will need to be repeated in other settings. Fourth, there was a substantial delay between the time of the original treatment decision and the time of the heart team review, which may create questions regarding whether evolving data were associated with changes in decisions. However, by 2012, the implications of the most important contemporary prognostic indicators with consequences for treatment decisions (ie, the presence of diabetes, left ventricular dysfunction, and anatomical complexity, as measured by SYNTAX score) were already known.^21,41,42^

## Conclusions

To our knowledge, this study is the first to suggest that heart team treatment recommendations may be different than those of the original treating interventional cardiologist in up to 30% of cases. This subset of cases was associated with more divergent opinions within the heart team and between interventional cardiologists. Moreover, when the heart team disagreed with the original treating interventional cardiologist, the original treatment was more frequently PCI or medication therapy and less frequently CABG, which may suggest that the presence of competing risks and benefits may underlie considerations of alternate treatment recommendations. Whether heart team reviews are associated with improvements in clinical outcomes, how heart team recommendations can best be used, and how patients should be selected for review a priori are questions that need to be further examined.

## Notes

Supplement. **eMethods.** Structured Template of Virtual Heart Team Case Presentation
