# Supplementary material for: Comparison of Heart Team vs Interventional Cardiologist Recommendations for the Treatment of Patients With Multivessel Coronary Artery Disease
Source: JAMA Netw Open. 2020 Aug 10;3(8):e2012749. doi: 10.1001/jamanetworkopen.2020.12749 (PMC7417969; doi:10.1001/jamanetworkopen.2020.12749)
Supplement: Supplement. — eMethods. Structured Template of Virtual Heart Team Case Presentation [file jamanetwopen-3-e2012749-s001.pdf]

Supplementary Online Content

Tsang MB, Schwalm JD, Gandhi S, et al. Comparison of heart team vs interventional cardiologist recommendations for the treatment of patients with multivessel coronary artery disease. *JAMA Netw Open*. 2020;3(8):e2012749. doi:10.1001/jamanetworkopen.2020.12749

eMethods. Structured Template of Virtual Heart Team Case Presentation

This supplementary material has been provided by the authors to give readers additional information about their work.

© 2020 Tsang MB et al. *JAMA Network Open*.

eMethods. Structured Template of Virtual Heart Team Case Presentation

Case Template

**Age**	**BMI**	**Gender**	**Patient Type**	Inpatient vs Outpatient

**Indication for Procedure**	
**Rest ECG: Ischemic Changes at rest**	
**Exercise ECG**	
**Functional Imaging**	

Catheterization Date:

Clinical History (including clinical course, electrocardiogram changes and troponin rise as relevant):

Electrocardiogram:

Echocardiogram:

Stress Test/ Perfusion Imaging:

Risk Factors:

Past Medical History / Co-morbidities:

Medications on Presentation to Heart Investigation Unit:

Allergies:

Social History:

**Social Situation:** (living situation, social supports, marital status, complexities with spouse or dependents)	
**Occupation:**	
**Drug Coverage:**	
**Functional Status** (independence with activities of daily living, mobility	

Physical Examination:

Relevant Blood Work:

© 2020 Tsang MB et al. *JAMA Network Open*.

**Hemoglobin**	
**INR**	
**Platelets**	
**Potassium**	
**Sodium**	
**Creatinine**	

Cardiac Catheterization
Aortic Pressure:
Left Ventricular Pressure:
Left Ventricular End Diastolic Pressure:

Left Ventricular Angiogram (Function, wall motion abnormalities, mitral regurgitation, Pull back gradients across aortic valve):

LEFT MAIN:

LEFT ANTERIOR DESCENDING:

RAMUS:

LEFT CIRCUMFLEX:

RIGHT CORONARY ARTERY:

**Euroscore**	% mortality
**STS Score**	% mortality<br>% morbidity and mortality
**Syntax Score**	

© 2020 Tsang MB et al. *JAMA Network Open*.
